# The effect of individual state on the strength of mate choice in females and males

**DOI:** 10.1093/beheco/arac100

**Published:** 2023-02-23

**Authors:** Liam R Dougherty

**Affiliations:** Department of Evolution, Ecology and Behaviour, University of Liverpool, Crown Street, Liverpool L69 7RB, UK

**Keywords:** condition dependence, life history, mate choice, mating preference, sexual selection, systematic review, terminal investment

## Abstract

Animals are thought to gain significant fitness benefits from choosing high-quality or compatible mates. However, there is large within-species variation in how choosy individuals are during mating. This may be because the costs and benefits of being choosy vary according to an individual’s state. To test this, I systematically searched for published data relating the strength of animal mate choice in both sexes to individual age, attractiveness, body size, physical condition, mating status, and parasite load. I performed a meta-analysis of 108 studies and 78 animal species to quantify how the strength of mate choice varies according to individual state. In line with the predictions of sexual selection theory, I find that females are significantly choosier when they are large and have a low parasite load, thus supporting the premise that the expression of female mate choice is dependent on the costs and benefits of being choosy. However, female choice was not influenced by female age, attractiveness, physical condition, or mating status. Attractive males were significantly choosier than unattractive males, but male mate choice was not influenced by male age, body size, physical condition, mating status, or parasite load. However, this dataset was limited by a small sample size, and the overall correlation between individual state and the strength of mate choice was similar for both sexes. Nevertheless, in both males and females individual state explained only a small amount of variation in the strength of mate choice.

## Teaser

Does being choosy about mates vary with an individual animal’s state? I performed a meta-analysis of 108 studies and 78 animal species testing how an individual’s state (age, attractiveness, body size, physical condition, mating history, or parasite load) influenced the strength of mate choice. Females show stronger mating preferences when they are large, and have few parasites, and attractive males are choosier. Future mate choice experiments should take into account differences in individual state or risk obtaining invalid results.

## INTRODUCTION

Across the animal kingdom, individuals of both sexes are often selective in who they choose to mate with (Rosenthal 2017). Mate choice is thought to be so widespread because it often provides choosers with reproductive benefits in terms of more or higher-quality offspring (Andersson 1994; Rosenthal 2017). However, empirical studies show that the strength of mate choice, which is the degree to which individuals prefer some mating options over others (Reinhold and Schielzeth 2015), can vary widely between species, between individuals of the same species, and even within an individual (reviewed in Jennions and Petrie 1997; Ah-King and Gowaty 2016; Rosenthal 2017). Understanding how such variation arises is important, because mate choice is both a key determinant of individual reproductive fitness and a driver of evolution by sexual selection (Jennions and Petrie 1997). A key outstanding question is the degree to which variation is driven by nonadaptive processes, such as limitations on the ability to select the best available partners (Rosenthal 2017; Dougherty 2020), or adaptive changes in relation to the fitness benefits of pursuing different reproductive strategies. Some proportion of the observed variation in the strength of mate choice may be explained by the fact that mate choice is inherently costly, because mate sampling requires time and energy (Sullivan 1994; Vitousek et al. 2007), and because choice requires the rejection of some acceptable partners, potentially reducing fecundity (Kokko and Mappes 2005; Greenway et al. 2015). This may mean that animals can maximize their reproductive fitness, by being selective during mating only when the benefits of choice outweigh the costs. The environment in which an individual samples potential mates has the potential to strongly influence the costs and benefits of choice (Jennions and Petrie 1997; Dougherty 2021a). For example, in some environments mate sampling may be energetically difficult, or lead to a greatly increased predation risk, and here individuals are expected to become less choosy (Magnhagen 1991; Hughes et al. 2012). A recent meta-analysis examining context-dependent mate choice found that animals exhibit significantly stronger mate choice when predation risk is low and there is a high density of potential mates to choose from (Dougherty 2021a). However, the average correlation seen in these studies was quite weak (Dougherty 2021a). Additionally, within-species variation in mate choice is often seen under controlled experimental conditions, in which individuals are expected to experience a similar environment. This suggests that there may be other important factors influencing the expression of mate choice.

The other key factor affecting the costs and benefits of being choosy is an individual’s own phenotype (Jennions and Petrie 1997; Gray 1999; Cotton et al. 2006; Dougherty 2021b), which is often referred to as their “individual state” (e.g., Sih et al. 2015; Jolles et al. 2020). Important aspects of an individual’s state include physical factors such as body size and physical condition, as well as life-history factors such as age and mating history. Individual state could influence the costs and benefits of being choosy in three ways. First, individual state may influence the energetic resources available for reproduction. When energetic resources are low, individuals need to prioritize basal metabolic processes and behaviors relating to survival at the expense of reproduction (Williams 1966; Stearns 1992; Duffield et al. 2017). The resources available for reproduction are reduced in animals that are malnourished or in poor physical condition (Cotton et al. 2006). Individuals with more parasites also typically have fewer resources to invest into reproduction, either because of the direct effect of parasites or costly upregulation of the host immune system (Cotton et al. 2006; Duffield et al. 2017). Resource level may also change as animals age, though the patterns are less straightforward (Kokko 1997; Umbers et al. 2015): in some species resource level seems to increase linearly with age (especially in species with indeterminate growth), while in others resources may peak at some intermediate age. Second, an individual’s state may influence their future reproductive potential, or “residual reproductive value.” When residual reproductive value is low, the importance of short-term mating success and the cost of rejecting potential mating partners increases (Williams 1966; Duffield et al. 2017). Residual reproductive value is affected by an individual’s lifespan/mortality risk, and so is often lowered for individuals that are old, in poor physical condition and highly parasitized (Cotton et al. 2006). It is also affected by an individual’s ability to acquire mates, and so may be lower for individuals that are small, in poor physical condition, highly parasitized, and who produce secondary sexual signals that are unattractive. Third, individual state influences the risk of failing to mate. For unmated individuals, rejecting a potential mate is risky, because there is always a chance that no other suitable mate will be found before death. Such a risk does not exist for mated individuals. This leads to the prediction that unmated individuals should exhibit reduced choosiness in order to achieve their first mating and ensure at least some reproductive fitness (Kokko and Mappes 2005; Tanner et al. 2019).

Sexual selection theory predicts that individuals should be choosier during mate choice when: 1) they have more resources to invest into mate assessment, so that they are more able to make accurate choices (Cotton et al. 2006; Ah-King and Gowaty 2016), 2) they have a high residual reproductive value, so they can afford to delay mating and wait for high-quality partners (Cotton et al. 2006; Ah-King and Gowaty 2016), or 3) they have already produced at least some offspring, so there is no risk of dying without mating (Kokko and Mappes 2005; Tanner et al. 2019). While mate choice theory was often first formulated assuming that females are the choosy sex, there is no reason why these predictions do not also apply to male mate choice, as long as reproduction is costly for males, and males benefit from choosing high-quality females. This means that both males and females are generally predicted to be choosier when they are attractive (possessing traits that make them more likely to be chosen as a mating partner), young, large, in good physical condition (having large energetic resources available for reproduction), mated, and have a low parasite load (Jennions and Petrie 1997; Cotton et al. 2006; Ah-King and Gowaty 2016). In many cases, these predictions are borne out by the empirical evidence. For example, studies have found that males are choosier when they are attractive (Bakker and Rowland 1995), and individuals of both sexes are choosier when they are young (Gray 1999; Kodric-Brown and Nicoletto 2001; Dukas and Baxter 2014), large (Amundsen and Forsgren 2003; Kuczynski et al. 2017), in good condition (Wearing-Wilde 1996; Bakker et al. 1999; Hunt et al. 2005), mated (Lynch et al. 2005; Iglesias-Carrasco et al. 2019), and have a low parasite load (López 1999; Mazzi 2004). However, other studies find significant effects in the opposite direction to that predicted by theory. For example, in some cases females in poor physical condition are choosier than those in good condition (e.g., Fisher and Rosenthal 2006; Immonen et al. 2009; Perry et al. 2009; Griggio and Hoi 2010), or small females are choosier than large females (Tudor and Morris 2009a; Robinson and Morris 2010).

The empirical data on state-dependent mate choice have been summarized in several narrative reviews (Cotton et al. 2006; Ah-King and Gowaty 2016; Kelly 2018). However, there has been only one attempt to quantitatively and systematically synthesize this large literature, and only in males. Pollo et al. (2021) recently examined the extent to which male age, body size, and condition influence male mate choice, using a sample of 60 studies. The authors found that males that were large or had a high physical condition exhibited stronger choices than males that were small or had a poor physical condition, but that male age did not influence the strength of male mate choice. However, this study did not examine other potentially important aspects of individual state such as attractiveness, mating status, or parasite load. The study also did not examine the larger literature on state-dependent choice in females. This means that we have no clear picture of whether male mate choice is more or less state-dependent than female mate choice. As stated above, sexual selection theory predicts that state-dependent changes in the strength of mate choice are driven by changes in the costs and benefits of being choosy, and these costs and benefits should always be present to some extent for both sexes. However, if the magnitude of the costs and/or benefits of being choosy differs between the sexes, this should lead to a concurrent change in the extent of state-dependent choosiness. For example, females typically invest more resources into offspring growth and survival (Trivers 1972), and benefit less from mating with multiple partners (Andersson 1994; Janicke et al. 2016), both of which lead to females typically having stronger mating preferences than males (Andersson 1994; Rosenthal 2017). However, whether these differences lead to concurrent sex differences in state-dependent mate choice is less clear.

Understanding how individual state influences mate choice is important for several reasons. First, it provides insights into how animals balance the trade-off between survival and reproduction. Second, it will improve our understanding of the ecological context of mate choice, because the environment has the potential to influence state factors such as attractiveness, body size, condition, and parasite load. Identifying these links will improve our understanding of the conditions favoring the evolution of mate choice, their expression in natural populations, and the population-level consequences of environmental change on sexual selection and population fitness (Cally et al. 2019). Third, it will allow us to identify aspects of individual state that need to be controlled or accounted for when performing mating experiments (Dougherty 2020). In order to quantify the extent of state-dependent mate choice across the animal kingdom, I systematically searched the literature for studies relating the strength of mate choice in male or female animals to one of six aspects of individual state: age, attractiveness (any aspect of chooser phenotype that functions to signal mate quality), body size, physical condition (any aspect of chooser phenotype that reflects the energetic resources available for reproduction), mating status, and parasite load. I then used phylogenetically controlled meta-analyses to quantify how differences in these six state factors relate to differences in the strength of male and female mate choice across the animal kingdom. I analyzed this using a hierarchical approach. First, I tested the extent to which the strength of mate choice is state-dependent overall, by combining estimates from all six state factors into a single analysis. I then examined the extent to which each state factor influences the expression of mate choice in isolation. I perform separate analyses for males and females, because the costs and benefits associated with mating and mate choice are likely to differ between the sexes. I predicted that both males and females will be most choosy when they are attractive, large, in good condition, and have a low parasite load, because such individuals tend to have both more resources to invest into reproduction and a higher residual reproductive value; and when mated, because unmated individuals have an incentive to mate indiscriminately in order to remove the risk of dying without mating (Table 1). I had no clear prediction for the relationship between choosiness and age because of the expected variability in age-dependent resource levels and residual reproductive value. My analysis differs from the recent study by Pollo et al. (2021) in two key ways. First, Pollo et al. (2021) compared mate choice of males assigned to groups based on their phenotype. Here, I instead focus on correlations between individual state and mate choice, allowing me to include all relevant phenotypic variation and avoid having to assign to a value to male or female traits. Second, I consider both male and female mate choice.

**Table 1:** Outline of how the six state factors investigated in the current study are predicted to relate to individual resource level, residual reproductive value, and the risk of dying without mating, as well as the coded effect size direction for each factor

State factor	Resources greatest when:	RRV is highest when:	Risk of dying without mating absent when:	Correlation positive when:
Age	Depends	Depends	—	Young
Attractiveness	Attractive	Attractive	—	Attractive
Body size	Large	Large	—	Large
Condition	Good condition	Good condition	—	Good condition
Mating status	—	—	Mated	Mated
Parasite load	Few parasites	Few parasites	—	Few parasites

Note the unclear predictions for age.

## METHODS

### Literature searches

I searched for relevant papers in two ways. First, I obtained all papers cited by two reviews of state-dependent mate choice: Cotton et al. (2006) and Ah-King and Gowaty (2016). I also searched for all studies citing these two reviews on the 13/08/2019. Second, I performed keyword searches using the online databases Web of Science and Scopus on the 13/08/2019. These searches were part of a larger project on state-dependent mating behavior, part of which has already been published (Dougherty 2021b); as such, the search terms were deliberately broad. I used the following search string relating to individual state: age OR “mated status” OR “mating status” OR “mating history” OR “number of matings” OR virgin* OR parasite* OR disease OR diet OR hunger OR food OR stress OR condition OR size OR weight OR quality OR attractiveness OR resource* OR “territory quality” OR “reproductive cycle” OR “social rank” OR inbreeding OR personality OR boldness OR exploration OR “behavioural syndrome*,” NOT human. I performed four separate searches using this string and one of four search strings relating to mating behavior:

1. (mate OR mating) AND (choice OR preference* OR choosiness OR rejection)
2. courtship OR courting OR “sexual signalling”
3. mate AND (sampl* OR search*)
4. “species recognition” OR “mate recognition” OR “reproductive isolation” OR (conspecific* AND discriminat*) OR ((mate OR mating) AND (hybridization OR reinforcement)) OR (mating AND (heterospecific* OR interspecific*))

The search and study selection processes are outlined in Supplementary Figure S1. All searches combined (all four search strings) resulted in 32 320 results. I then screened the titles to remove obviously irrelevant studies (e.g., studies on humans, other subjects, review articles; Supplementary Figure S1). This resulted in 7158 studies. I then screened all relevant abstracts using the Rayyan website (Ouzzani et al. 2016). This resulted in 1230 promising studies, which were downloaded and read in order to test against my inclusion criteria. For this study, I successfully extracted data from 108 studies: 82 studies provided data for females (Archard et al., 2006; Atwell & Wagner, 2014; Atwell & Wagner, 2015; Auld et al., 2016; Bakker et al., 1999; Bastien et al., 2018; Bolund et al., 2010a; Bolund et al., 2010b; Borg et al., 2006; Brandt et al., 2005; Buchholz, 2004; Burley & Foster, 2006; Burley & Moran, 1979; Carrière & McNeil, 1990; Chen et al., 2018; Choudhury & Black, 1993; Churchill et al., 2019; Cordoba-Aguilar et al., 2003; Dakin & Montgomerie, 2014; Farrell et al., 2015; Fisher & Rosenthal, 2006; Gray, 1999; Griggio & Hoi, 2010; Guevara-Fiore et al., 2010; Hamilton & Poulin, 1999; Havens et al., 2011; Head et al., 2017; Hernandez-Jimenez & Rios-Cardenas, 2017; Honarmand et al., 2015; Hopwood et al., 2016; Hunt et al., 2005; Iglesias-Carrasco et al., 2017; Jennions et al., 1995; Joyce et al., 2009; Judge et al., 2014; Klein et al., 2012; Kodric-Brown & Nicoletto, 2001; Krishna & Hegde, 2003; Lerch et al., 2011; Ligout et al., 2012; López, 1999; Luck & Joly, 2005; Lynch et al., 2005; Lyons et al., 2014; Magallon-Gayon et al., 2011; Mair & Blackwell, 1998; Maksimowich & Mathis, 2001; Manrique & Lazzari, 1994; Mazzi, 2004; Mazzi et al., 2004; Mellan et al., 2014; Michaelidis et al., 2006; Milner et al., 2010; Morris et al., 2006; Morris et al., 2010; Moskalik & Uetz, 2011; Perry et al., 2009; Pfennig et al., 2013; Pfennig & Tinsley, 2002; Place et al., 2014; Poulin, 1994; Ptacek & Travis, 1997; Riebel et al., 2009; Ringo et al., 2005; Rios-Cardenas et al., 2007; Robinson & Morris, 2010; Rodriguez & Greenfield, 2003; Schmidt et al., 2013; Sisodia & Singh, 2004; Somashekar et al., 2011; Sommer-Trembo et al., 2016; Suk & Choe, 2008; Syriatowicz & Brooks, 2004; Tudor & Morris, 2009a; Villarreal et al., 2018; Vitousek & Romero, 2013; Webberley et al., 2002; Wong et al., 2011; Woodgate et al., 2010; Woodgate et al., 2011; Zeh & Zeh, 2007; Zuk et al., 1998) and 33 studies provided data for males (Amundsen & Forsgren, 2003; Bakker & Rowland, 1995; Barry, 2013; Bierbach et al., 2015; Burley & Moran, 1979; Chen et al., 2018; Choudhury & Black, 1993; Foote, 1988; Fox et al., 2019; Gaskett et al., 2004; Hedrick & Kortet, 2012; Heubel & Schlupp, 2008; Hoefler, 2007; Holveck et al., 2011; Iglesias-Carrasco et al., 2019; Kavaliers et al., 1997; King et al., 2005; Kvarnemo & Simmons, 1998; Lemaitre et al., 2012; Ludlow & Itzkowitz, 2007; Mazzi, 2004; Mellan et al., 2014; Muraco et al., 2014; Murphy, 1980; Ptacek & Travis, 1997; Schneider et al., 2016; Takahashi & Watanabe, 2010; Takahashi & Watanabe, 2011; Thunken et al., 2011; Tudor & Morris, 2009b; Villarreal et al., 2018; Wada et al., 2011; Wearing-Wilde, 1996).

### Inclusion criteria

In order to be eligible for inclusion, a study had to report within-species variation in mate choice in relation to one or more of the six state factors listed above. I included data on any animal species, with the exception of humans and species without fixed reproductive roles (hermaphrodites). Studies also had to provide enough information to allow me to calculate an appropriate effect size. I considered only precopulatory mate choice, because of the difficulty in attributing postmating outcomes to either sex. I therefore excluded studies examining peri- and postcopulatory behaviors such as ejaculate allocation, copulation duration, mate guarding, and parental investment. I included both experimental and correlative data, from both lab and field studies, but excluded studies in which multiple state factors were confounded (e.g., Coleman et al. 2004: older females were also significantly heavier). I included studies in which individuals were able to choose between members of the opposite sex (hereafter “courters”) or any other sexual signals (e.g., models, videos, scent marks, songs: Dougherty 2020). I included studies in which stimuli were presented sequentially or simultaneously (Dougherty and Shuker 2015; Dougherty 2020). I also included studies examining mate choice with respect to species identity, because a few studies have shown that the strength of mate choice for conspecifics over heterospecifics can also be state dependent (Pfennig et al. 2013; Schmidt et al. 2013; Willis 2013).

In order to facilitate the calculation of an effect size, I used a broad definition of the strength of mate choice, incorporating three types of mating behavior. First, I included studies that recorded how chooser behavior was targeted at some courter traits or sexual signals over others. Common behaviors included in this category were association time, approach or approach latency, and number of courtship or receptive displays. For these studies I used the measure of “choosiness” formulated by Reinhold and Schielzeth (2015, and see Dougherty 2021a). Here, the strength of choice was defined as the degree to which chooser behavior was nonrandom with respect to a given range of courter traits or sexual signals. Therefore, the greater the bias toward some traits or sexual signals, the “stronger” the choice, and the choosier an individual is. For example, for a two-choice test, the greater the bias toward one stimulus over another, the stronger the choice. For an observational study examining natural variation in courter traits, the stronger the correlation between the trait and chooser behavior, the stronger the choice. Focusing on the extent to which chooser behavior is nonrandom, without for example specifying the shape of a preference, allows me to incorporate many different types of choice data. Importantly, this framework allows me to convert a measure of statistical difference into a correlation coefficient (see “Effect sizes” section). However, I note that this approach does not match the accepted definition of choice strength that is obtained using a preference function approach (e.g., Kilmer et al. 2017). This is because restricting myself to species with preference function data would have reduced the sample size greatly. Second, I included studies that recorded active rejection behaviors resulting from a mating attempt by a courter (e.g., Kvarnemo and Simmons 1998; Perry et al. 2009). I assumed that individuals who rejected more mating attempts had a stronger mating preference. Third, I included studies that recorded the number of mates visited by choosers (e.g., Choudhury and Black 1993; Dakin and Montgomerie 2014). I assumed that choosers that sample more mates before making a choice had a stronger mating preference.

Differences in some aspects of individual state may be detectable by courters, leading to changes in courter behavior. For example, males may increase their courtship effort toward large females compared with small females. This could result in an apparent increase in female choice that is entirely driven by changes in male behavior. I attempted to reduce this problem by excluding studies in which courters could physically interact with choosers, and so potentially coerce them into mating (99 studies in total were excluded for this reason). This meant that I excluded most studies that used mating outcomes to infer mate choice. The exception to this were those species in which active participation in mating by choosers can be inferred. For example, I included two examples of male choice in species where males mount females during mating (Murphy 1980; King et al. 2005), as here females do not coerce males to mate. However, this approach does not completely rule out courter influence on choice outcomes; for example, courters could change their behavior when behind a partition in a way that could influence chooser behavior. I therefore also tested for the influence of courter interference by comparing effect sizes between studies in which behavioural interactions between choosers and courters were possible, and studies in which choosers were presented with sexual stimuli in the absence of live mates (see “Moderators” section).

### Factors and predictions

#### Age

I included age-dependent effects as long as all individuals were mature (not juveniles). Importantly, age is often confounded with mating status in natural populations. To exclude this possibility, I only considered studies testing unmated individuals. I included both studies in which age could be measured precisely (as in lab studies) and studies in which age was estimated (e.g., using morphological indicators). I had no clear prediction for the relationship between choosiness and age, because of the expected variability in age-dependent resource levels and residual reproductive value (Table 1).

#### Attractiveness

I define attractiveness as any aspect of chooser phenotype or behavior that could signal mate quality, and does not fall into any of the other categories listed. There were four main traits in this category. For females, I included inbreeding level and social rank. For males, I included social rank, ornament color, and nest quality. Studies of social rank had to test mate choice in the absence of rivals, as dominant individuals could potentially control subordinate mating behaviors. I predicted that attractive individuals would be choosier than unattractive individuals (Table 1), as the former are expected to have more available resources to invest into reproduction and will typically receive a greater number of mating opportunities.

#### Body size

This category included measures of body weight, body length, and other proxy measures of body size (e.g., leg or wing length, carapace width). I predicted that large individuals would be choosier than small individuals (Table 1), because the former are expected to have more resources available to invest into reproduction and are more competitive, so will generally have more mating opportunities.

#### Condition

I define individual condition following Rowe and Houle (1996), as “the pool of resources acquired from the environment, which is available for allocation to various fitness-related traits.” This category included studies that compared individuals that differed in: 1) exposure to different food levels or dietary nutritional content, 2) early-life diet or the number of siblings during rearing (brood size), 3) exposure to environmental stressors that cause increased physiological costs (oxygen levels or environmental pollutants in aquatic animals), 4) body weight relative to body length, and 5) movement ability following experimental wing clipping in birds. I assumed that individuals were in poor condition if they had experienced low food levels, poor-quality diets or stressful environments, were relatively light for a given body size, or had their movement ability experimentally reduced. I note that the extent to which some of these indices of individual condition (notably the morphological measures) actually reflect available energy reserves has been criticized (Clancey and Byers 2014; Wilgers and Hebets 2015). I predicted that individuals in good condition would be choosier than those in poor condition (Table 1), because individuals in good condition have more resources available to invest into reproduction, and tend to be more attractive, more competitive, live longer, and receive more mating opportunities.

#### Mating status

This category included studies comparing mate choice between unmated and once-mated individuals. I assume that for once-mated individuals the mating resulted in successful reproduction. I did not include studies comparing mate choice in relation to the number of matings above one. I predicted that mated individuals would be choosier than unmated individuals (Table 1), because unmated individuals have an incentive to mate indiscriminately in order to remove the risk of dying without mating.

#### Parasite load

This category included any measurement of the number of internal or external parasites. I excluded vertically transmitted endosymbionts (e.g., Wolbachia in insects) because these associations can be complex, ranging from mutualistic to pathogenetic, and fitness costs to hosts are rarely measured (Moran et al. 2008). I also excluded studies inducing an immune challenge using inactivated pathogens or other factors. This is because the immune response is often short lived, and with a defined peak, so that detecting differences in host behavior strongly depends on which point of the immune response curve individuals are sampled at. I predicted that individuals with a low parasite load would be choosier than those with a high parasite load (Table 1), because the former have more resources available to invest into reproduction and tend to be more attractive, more competitive, live longer, and receive more mating opportunities.

### Effect sizes

I used the correlation coefficient *r* as the effect size in this analysis. Here, *r* represents the extent to which the strength of choice varies between individuals in relation to one of the six state factors listed above. For all analyses, I used Fisher’s *Z* transform of the correlation coefficient (*Zr*) as the response variable, as this adheres better to a Gaussian distribution at high magnitudes (Koricheva et al. 2013). The associated variance for *Zr* was calculated as 1/(*n* − 3) (Borenstein et al. 2009), with n being the total number of animals used in the test. The correlation coefficient can take values between −1 and +1. I arbitrarily assigned correlations a positive direction when individuals predicted to have a high residual reproductive value were choosier than those predicted to have a low value. Therefore, correlations were coded as positive when individuals were choosier when they were young, attractive, large, in good condition, mated, or had a low parasite load. Correlations were coded as negative when individuals were choosier when they were old, unattractive, small, unmated, shy, in poor condition, or had a high parasite load (Table 1). This coding scheme means that I generally predict that the overall mean correlation will be positive for both sexes.

In several cases, studies often reported nonsignificant results without describing the direction of the effect. These data points are traditionally excluded from meta-analyses. However, this systematically biases the dataset against the inclusion of nonsignificant results (Harts et al. 2016). In order to address this problem, I assigned them a value of zero, and ran the analyses with and without including these extra data points. I refer to these data points as “directionless data points.” This resulted in two separate datasets: a full dataset including directionless data points, and a reduced dataset with directionless data points excluded.

I obtained effect sizes by: 1) obtaining correlations reported in the text or tables, 2) using presented data or statistical tests to calculate a correlation myself, or 3) re-analyzing raw data presented in the paper or the Supplementary Material. When studies compared mate choice between two categories or treatments, I first calculated the standardized mean difference (Hedges’ *d*: Koricheva et al. 2013), which was then converted into *r*. For studies comparing mating behavior targeted at different courters or sexual signals, I calculated effect sizes in two ways. For frequency data, such as the numbers of choices for one option over another, an effect size could be calculated from a 2 × 2 frequency table, tallying the number of choices in each context. For continuous data, such as the time spent in association with a courter, preferences had to be presented as a single value, such as a difference score (the difference in preference between the preferred and the nonpreferred option) or some other metric, to facilitate comparison across contexts. In summary, I calculated effect sizes using one of four types of data: correlations (*N* = 34 for females and 19 for males), statistical tests comparing two groups (*N* = 26 for females and 5 for males), means and variances for two groups (*N* = 59 for females and 17 for males), and frequency data (*N* = 23 for females and 18 for males). I therefore also performed a sensitivity analysis to check for any systematic differences in the mean effect size between these four types of data, and found none for males or females (see Supplementary Results for details).

### Moderators

For each correlation, I additionally recorded information relating to four potential moderators of state-dependent mate choice:

1. *Taxonomic group*. I predicted that mate choice would be most state dependent for members of relatively long-lived groups (e.g., mammals, birds, amphibians) compared with members of short-lived groups (e.g., insects, arachnids). This is because life-history theory predicts that short-lived species will benefit more by investing maximally into reproduction irrespective of individual state.
2. *State factor*. I compared whether the extent of state-dependent mate choice depended on which of the six state factors was examined. As discussed below, I predicted that choosy individuals were more likely to be young, attractive, large, mated, in good condition, and have a low parasite load. I also predicted that body size, physical condition, and parasite load should have the strongest influence on mate choice, because these factors have the potential to greatly influence resource levels, competitiveness, and expected future mating opportunities.
3. *State variation*. I recorded whether individual state varied naturally or was experimentally manipulated. I predicted that choice would be most state dependent when a state factor was experimentally manipulated, as this should increase the ability to statistically detect between-group differences.
4. *Courter interaction*. I recorded the extent to which males and females could interact during mating trials. This resulted in three categories: 1) males and females could physically interact, 2) males and females could interact behaviorally but could not physically touch each other, or 3) live mates were not present during mate choice tests. If courters behave differently toward choosers of different states, then I predict that mate choice will be more state dependent in categories 1) and 2), as interaction between courters and choosers is not possible in category 3).

### Phylogeny

In order to account for the potential nonindependence of correlations from closely related species, I constructed a supertree for each dataset using the Open Tree of Life (OTL) database (Hinchliff et al. 2015). Trees were created in R v4.0.3, using the Rotl (Michonneau et al. 2016) and Ape (Paradis et al. 2004) packages. For cases where the OTL database resulted in polytomies, I manually searched for published phylogenetic trees for the branches in question. For the relationships among the genus *Gryllus* I used (Huang et al. 2000). For the relationships among the family *Gryllidae* I used (Chintauan-Marquier et al. 2016). For the relationships among the genus *Xiphophorus* I used (Cui et al. 2013). Given the absence of accurate branch length data for these trees, branch lengths were first set to one and then made ultrametric using Grafen’s method (Grafen 1989). For analyses including subsets of the data, I used an appropriately pruned tree. The final tree can be seen in Supplementary Figure S2.

### Statistical analysis

All analyses were performed using R v4.0.3 (R Development Core Team 2020), and the package metafor v2.4 (Viechtbauer 2010). I performed all analyses separately for males and females. For each sex, I first ran an intercept-only multilevel random-effects model using the rma.mv function in Metafor, in order to determine the overall mean correlation including all six state factors (in other words, the extent to which the strength of mate choice is state dependent). These models included Fisher’s *Z* transform of the correlation coefficient (*Zr*) as the response variable, weighted by study sampling variance, and included phylogeny, species, study ID, and observation ID as random factors. Phylogeny was incorporated into the model using a correlation matrix, assuming that traits evolve via Brownian motion. An observation-level random factor (observation ID) is required to correctly estimate residual heterogeneity (Moran et al. 2020). The mean correlation was considered to be significantly different from zero if the 95% confidence intervals did not overlap zero. For each sex I ran this model using the full dataset, and then after excluding directionless data points.

I used two methods to estimate effect size heterogeneity. First, I calculated *I*^2^, which is the proportion of variance in effect sizes which is not attributable to sampling (error) variance (Higgins et al. 2003). I used the method of Nakagawa and Santos (2012) to partition heterogeneity among the random factors in the model. With this method I calculated total *I*^2^, as well as the heterogeneity explained by shared evolutionary history (phylogenetic *I*^2^), differences between species (species-level *I*^2^), differences between studies (study-level *I*^2^), and differences between data points (residual *I*^2^). *I*^2^ values of 25%, 50%, and 75% are considered low, moderate, and high, respectively (Higgins et al. 2003). However, total *I*^2^ values in excess of 75% are very common in the large, multispecies datasets seen in ecological and evolutionary meta-analyses (Senior et al. 2016). Second, I calculated 95% prediction intervals (95% PIs) using the predict function in metafor, following IntHout et al. (2016) and Nakagawa et al. (2021).

I investigated potential moderators of the correlation using the full dataset for each sex. Specifically, I used this approach to test how each of the six state factors influenced choosiness in isolation. To do this, I ran meta-regression models, which were identical to the above model except for the inclusion of one of the moderators as a (categorical or continuous) fixed effect. I ran a separate model for each moderator, and did not test for interactions between moderators. I determined significance using the *Q*_M_ statistic. In order to increase the robustness of the results from these models, I excluded any factor levels that had fewer than 10 data points. I also calculated the amount of variance explained by the fixed factor in each model (marginal *R*^2^) using the orchaRd R package (Nakagawa and Schielzeth 2013; Nakagawa et al. 2021). Finally, in order to determine the average correlation for each level of any categorical moderator, I ran the same meta-regression as above, but excluded the model intercept. This effectively runs a separate model for each moderator category, including those with fewer than 10 data points. Again, I ran a separate model for each moderator.

I searched for two signs of publication bias for males and females. First, I tested for a change in effect size over time, in relation to study publication year. Second, I searched for potential small-study effects by testing for a relationship between effect size (*Zr*) and the standard error. To do this I ran a minus-intercept meta-regression model with phylogeny, species, study ID, and observation ID as random factors, and mean-centered publication year and standard error as fixed effects, following Nakagawa et al. (2022). I then adjusted the mean effect size estimate for each dataset after taking publication bias into account, using a meta-regression with mean-centered publication year and study variance as fixed factors, including the intercept, following Nakagawa et al. (2022). I performed this adjustment regardless of whether either of these models detected a significant trend.

## RESULTS

### Females

**Figure 1:**
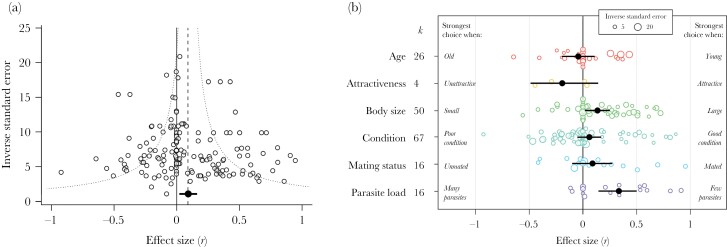
The female dataset. (A) Funnel plot showing the relationship between effect size (the correlation coefficient *r*) and the inverse standard error (a measure of study precision—larger values represent studies with larger sample sizes) for females (*k* = 179). The dashed line shows the overall mean effect size estimate from the meta-analysis model. The dotted line illustrates the typical expected “funnel” shape, with effect sizes from studies with large sample sizes resulting in estimates that are closer to the mean. (B) Orchard plot showing the mean effect size estimate (black circles), plus 95% confidence intervals, for each state factor separately. Open circles show the raw correlations, with the size of circles corresponding to study precision (inverse standard error). Annotations refer to the scheme used to assign correlations a positive or negative sign. *k* = the number of effect sizes for each category.

I obtained 179 correlations for females, from 82 studies and 62 species. I obtained data from 8 taxonomic groups, but most correlations were for insects (*k* = 69) and fish (*k* = 58). Of the six factors examined, the majority of correlations related to body size (*k* = 50) and condition (*k* = 67). Only four correlations considered female mate choice with respect to species identity. Overall, the mean correlation for females was significantly positive, suggesting that females exhibit stronger mate choice when they have more resources to invest into reproduction, a higher residual reproductive value, or the cost of rejecting potential mates is reduced (mean *r* = 0.09, 95% CI = 0.03 to 0.16, 95% PI = −0.51 to 0.64, *k* = 179; Figure 1A). Removing the 39 directionless data points led to a small increase in the mean correlation (mean *r* = 0.13, 95% CI = 0.03 to 0.22, 95% PI = −0.57 to 0.72, *k* = 140). The full dataset showed “high” total heterogeneity (total *I*^2^ = 87.5%), which is normal for multispecies ecological datasets (Senior et al. 2016). A low–moderate proportion of this heterogeneity was attributable to between-study differences (46%). Phylogenetic history and species explained a negligible amount of heterogeneity in *Zr* (<0.1% each), with the remaining 41.5% attributable to observation-level differences.

The degree of state-dependent variation in female mate choice depended on which state factor was measured (Table 2). In line with my predictions, females were choosier when they were large and had a low parasite load (Figure 1B; Supplementary Table S1). However, pairwise post hoc tests did not detect any significant differences in mean effect size between the five state categories with more than 10 effect sizes (Supplementary Table S2). There was no significant effect of female age, attractiveness, condition, or mating status on the strength of female mate choice when each factor was tested in isolation (Figure 1B; Supplementary Table S1). The degree to which mate choice was state dependent was not significantly influenced by taxonomic group, whether individual state was varied naturally or was experimentally manipulated, or the extent to which males and females could interact during mating trials (Table 2; Supplementary Table S1).

**Table 2:** Results from meta-regressions testing the six moderator variables for the female and male datasets

Moderator	Females				Males			
	*k*	Marginal *R*^2^	*Q*_M_	*P*	*k*	Marginal *R*^2^	*Q*_M_	*P*
Taxonomic group	155	0.01	0.58	0.75	47	0.137	2.66	0.10
State factor	175	0.08	9.8	0.04	60	0.003	0.14	0.93
State variation	179	<0.001	0.002	0.97	71	0.002	0.12	0.73
Courter interaction	179	0.004	0.33	0.85	71	0.006	0.37	0.83

Each moderator was tested using a separate meta-regression model. The *Q*_M_ statistic tests whether the moderator variable significantly influences the mean correlation. Marginal *R*^2^ is the amount of variance explained by each fixed factor. Significant moderators are highlighted in gray.

I found evidence for publication bias in the female choice dataset. The mean effect size decreased significantly over time (β = −0.01, *z* = −2.61, *P* < 0.001; Supplementary Figure S3). Additionally, studies with small sample sizes were more likely to report larger effects (β = 0.54, *z* = 2.64, *P* = 0.01; Supplementary Figure S4), and the overall adjusted mean effect size for females did not differ significantly from zero after taking both forms of publication bias into account (mean *r* = 0.04, 95% CI = −0.02 to 0.10, 95% PI = −0.53 to 0.72, *k* = 179).

### Males

**Figure 2:**
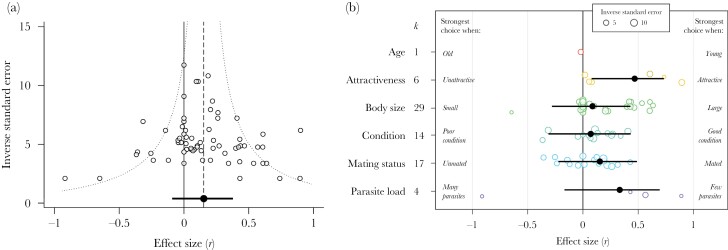
The male dataset. (A) Funnel plot showing the relationship between effect size (the correlation coefficient *r*) and the inverse standard error (a measure of study precision—larger values represent studies with larger sample sizes) for males (*k* = 71). The dashed line shows the overall mean effect size estimate from the meta-analysis model. The dotted line illustrates the typical expected “funnel” shape, with effect sizes from studies with large sample sizes resulting in estimates that are closer to the mean. (B) Orchard plot showing the mean effect size estimate (black circles), plus 95% confidence intervals, for each state factor separately. Open circles show the raw correlations, with the size of circles corresponding to study precision (inverse standard error). Annotations refer to the scheme used to assign correlations a positive or negative sign. *k* = the number of effect sizes for each category.

I obtained 71 correlations for males, from 33 studies and 29 species. I obtained data from 6 taxonomic groups, but most correlations were for insects (*k* = 18) and fish (*k* = 29). Of the six factors examined, the majority of correlations related to body size (*k* = 29), condition (*k* = 14), and mating status (*k* = 17). Only five correlations considered male mate choice with respect to species identity. Overall, individual state did not significantly influence the strength of male mate choice (mean *r* = 0.15, 95% CI = −0.09 to 0.38, 95% PI = −0.48 to 0.79, *k* = 71; Figure 2A). Removing the 12 directionless data points led to a small increase in the mean correlation, but it still did not differ significantly from zero (mean *r* = 0.18, 95% CI = −0.1 to 0.44, 95% PI = −0.48 to 0.72, *k* = 59). The full dataset showed high total heterogeneity (total *I*^2^ = 76.6%), with 34.4%, 24.1%, and 18.1% attributable to phylogenetic history, species-level differences, and observational-level differences, respectively. Study-level differences explained a negligible amount of heterogeneity in *Zr* (<0.1%).

The degree to which male mate choice was state dependent did not depend on which state factor was examined (Table 2). However, when considering the six state factors separately, as predicted, males were choosier when they were more attractive (Figure 2B). However, this category includes only six estimates. There was no significant effect of male age, body size, condition, mating status, or parasite load on the strength of male mate choice when each factor was tested in isolation (Figure 2B; Supplementary Table S2). The degree to which male mate choice was state dependent did not depend the taxonomic group, whether individual state was varied naturally or was experimentally manipulated, or the extent to which males and females could interact during mating trials (Table 2; Supplementary Table S3).

There was no significant relationship between the degree to which male mate choice was state dependent and the year of publication (β = 0.001, *z* = 0.183, *P* = 0.85; Supplementary Figure S3) or the standard error (β = 0.67, *z* = 1.37, *P* = 0.17; Supplementary Figure S4), and the overall adjusted mean effect size for males was slightly reduced after taking publication bias into account, still did not differ significantly from zero (mean *r* = 0.03, 95% CI = −0.11 to 0.17, 95% PI = −0.47 to 0.52, *k* = 71).

## DISCUSSION

Animals can improve their reproductive success by being prudent during mate choice. However, the benefits of being choosy are predicted to depend on an individual’s state, because of differences in their ability to pay the costs of reproduction, their expected number of future mating opportunities, and the risk of dying without mating. For these reasons, both males and females should be able to maximize their reproductive fitness by strategically altering the strength of mate choice in relation to their individual state. Using phylogenetically controlled meta-analysis, I tested how the strength of male and female mate choice is affected by individual age, attractiveness, body size, condition, mating status, and parasite load. When all six factors were considered together, female mate choice was significantly state dependent, and in the direction predicted by sexual selection theory: individuals are choosier when in a state that gives them more resources to invest into reproduction and a higher residual reproductive value. However, the average correlation was small, and adjusting this estimate after controlling for publication bias resulted in an overall estimate that was not significantly different from zero. Nevertheless, when the six state factors were considered separately, I found that females are significantly choosier when they are large and have a low parasite load. This suggests that some aspects of individual state influence the strength of female mate choice more than others. In contrast to female mate choice, male mate choice was not significantly influenced by individual state when all six factors were considered together. However, the male dataset was significantly smaller than the female dataset, thus resulting in reduced statistical power, and that the average correlation for the male dataset was actually slightly larger than that found in the female dataset. The assertion that the male dataset is underpowered is supported by the fact that Pollo et al. (2021) found that male state did significantly influence the strength of male mate choice, using a larger dataset of 53 studies. When considered separately, attractive males (males that were relatively outbred, produced high-quality nests, or possessed bright ornaments) exhibited stronger mate choice than unattractive males, however the small sample size of only six correlations mean it would be premature to draw a strong conclusion from this.

As predicted by sexual selection theory (Jennions and Petrie 1997; Cotton et al. 2006; Ah-King and Gowaty 2016), females exhibited significantly stronger mating preferences when they were large and had a low parasite load. Such individuals are expected to have increased resources and a higher residual reproductive value compared with individuals that are small or have a high parasite load, which suggests that this effect may be driven by changes in the costs and benefits of being choosy. Importantly, the fact that the type of courter interaction did not influence the degree of state-dependent mate choice is consistent with the interpretation that these changes are not being driven by changes in male display behavior toward females. I also predicted that females would exhibit stronger mate choice when young, attractive, unmated, and in good condition. However, the mean estimates for the age, mating status and condition categories did not differ significantly from zero (I did not include attractiveness in the meta-regression tests, because I only obtained four data points for this category). The lack of a significant age effect may be because the relationship between age and both resource level and residual reproductive value is complex, and likely varies depending on a species’ life-history strategy (Kokko 1997; Umbers et al. 2015). The lack of a significant effect of condition is surprising, given the important role that energy resources are predicted to play in life-history decisions relating to reproduction (Cotton et al. 2006). However, one potential explanation is that studies may often use convenient measures of state, such as mass/size indices, that only weakly relate to an individual’s resources or residual reproductive value (Clancey and Byers 2014; Wilgers and Hebets 2015). However, the majority of included studies experimentally manipulated condition by altering food levels or diet in ways that should have altered individual resources to some extent. Finally, the lack of a significant effect of mating status could be explained if the risk of dying unmated is generally low, or the benefits both mated and unmated females gain from being choosy outweigh this risk. Further, in polyandrous species, any factors favoring increased choosiness by unmated females may be balanced by the fact that these females may often receive more mating opportunities than mated females, from males trying to avoid sperm competition (Bonduriansky 2001).

The average correlation between individual state and the strength of mate choice was for 0.15 males and 0.08 for females, both of which are considered “small” based on common benchmarks (Cohen 1992). This primarily arises because of the high heterogeneity in the dataset (see below), and the large number of correlations close to zero. In other words, there are a large number of studies in both sexes for which the strength of mate choice in was unrelated to individual state. Despite this, I also detected evidence for publication bias in the female dataset, in that the average correlation between choosiness and individual state has decreasing significantly over time, and studies with small sample sizes are more likely to report significantly positive correlations. Notably, Pollo et al. (2021) also found a similar temporal trend in their dataset of state-dependent male choosiness. Additionally, for females the small average correlation can be partly attributed to the many negative correlations, corresponding to cases where mate choice was stronger for females that have fewer resources or a lower residual reproductive value. Such negative correlations were most common for the condition category. A major outstanding question is whether these negative correlations reflect sampling error or real biological patterns. One adaptive explanation is that negative correlations could arise when both choosers and courters differ in the same aspect of individual state, especially if choosers directly benefit from mate choice. For example, poor-condition females could benefit more than their high-condition rivals from choosing high-condition males (Fisher and Rosenthal 2006; Rosenthal 2017), especially in species where males provide food or water during mating (Johnson et al. 1999; Immonen et al. 2009). However, studies examining mate choice when both choosers and courters differed in either condition or parasite load were very rare (only three estimates in the female dataset and two in the male dataset), which suggests that this is not a general explanation for the large number of negative correlations obtained.

Both the male and female datasets were characterized by very high heterogeneity, and none of the moderator variables I tested explained more than 13% of the observed variance in *Zr* for either sex. This means that adaptive reaction norms driven by changes in the costs and benefits of being choosy explain only a small amount of observed variation in the strength of mate choice. Notably, a similar conclusion can be made when examining environment-driven changes in the strength of mate choice (Dougherty 2021a). This suggests that there are important moderating factors that remain unaccounted for. One important missing factor is the cost of choice. This is because all of the predictions relating to state-dependent mate choice rely on the assumption that being choosy is costly, and we lack estimates of these costs for most species (but see e.g., Byers et al. 2005; Vitousek et al. 2007; Torsekar et al. 2019). In fact, for many species it may be that the cost of expressing a mating preference is small, especially in relation to the time and energy spent sampling mates (Gibson and Bachman 1992; Wickman and Jansson 1997). This might often be true for females, for example because sperm can be stored for a long time (meaning that a small number of matings are sufficient to ensure lifetime fecundity), or because the correlation between the number of matings and reproductive output (the Bateman gradient) is less than one. Further, these costs could in turn be influenced by differences in a species’ environment or mating system (Dougherty 2021a). Alternatively, causal relationships could be obscured by the fact that many aspects of individual state are correlated. For example, large individuals are often more attractive than small individuals (Andersson 1994; Rosenthal 2017), and condition and parasite load are often tightly related (e.g., Beldomenico and Begon 2010). Finally, there are likely to be methodological differences between studies that I have not controlled for (Dougherty 2020). For example, Pollo et al. (2021) found that male mate choice was more strongly related to male quality when males had multiple options to choose from (see also Dougherty and Shuker 2015).

In summary, the present meta-analysis of 108 studies found that mate choice is significantly stronger for females that are large or have a low parasite load, and males that are attractive. Such individuals are expected to have increased resources to invest into reproduction, or a higher residual reproductive value, all of which suggests that this effect is driven by changes in the costs and benefits of being choosy. These results imply that both experimental and observational studies examining female (and possibly male) mate choice should consider controlling for differences in female health and body size, or run the risk of obtaining spurious results (Dougherty 2020). However, there were a range of other state factors that did not influence choosiness in one or both sexes. For the male dataset any null results may be partly the result of reduced statistical power. This is supported by the fact that the average correlation is actually larger for males than for females in this analysis, and that Pollo et al. (2021) found that male state did significantly influence the strength of male mate choice, using a larger dataset of 53 studies. Nevertheless, in both males and females individual state explained only a small amount of variation in the strength of mate choice, and both datasets were characterized by high heterogeneity which remains mostly unexplained even after taking into account differences in taxonomy, phylogeny, and experimental design.

## Supplementary Material

arac100_suppl_Supplementary_Material
