# Supplementary material for: The effect of individual state on the strength of mate choice in females and males
Source: Behav Ecol. 2023 Feb 23;34(2):197–209. doi: 10.1093/beheco/arac100 (PMC10047626; doi:10.1093/beheco/arac100)
Supplement: arac100_suppl_Supplementary_Material [file arac100_suppl_supplementary_material.docx]

Supplementary methods and results:

**The effect of individual state on the strength of mate choice in females and males**

Liam R. Dougherty

Department of Evolution, Ecology and Behaviour, University of Liverpool, Crown Street, Liverpool, L69 7RB, UK

E-mail: liam.dougherty@liverpool.ac.uk; Tel.: +44 0151 795 7771

**
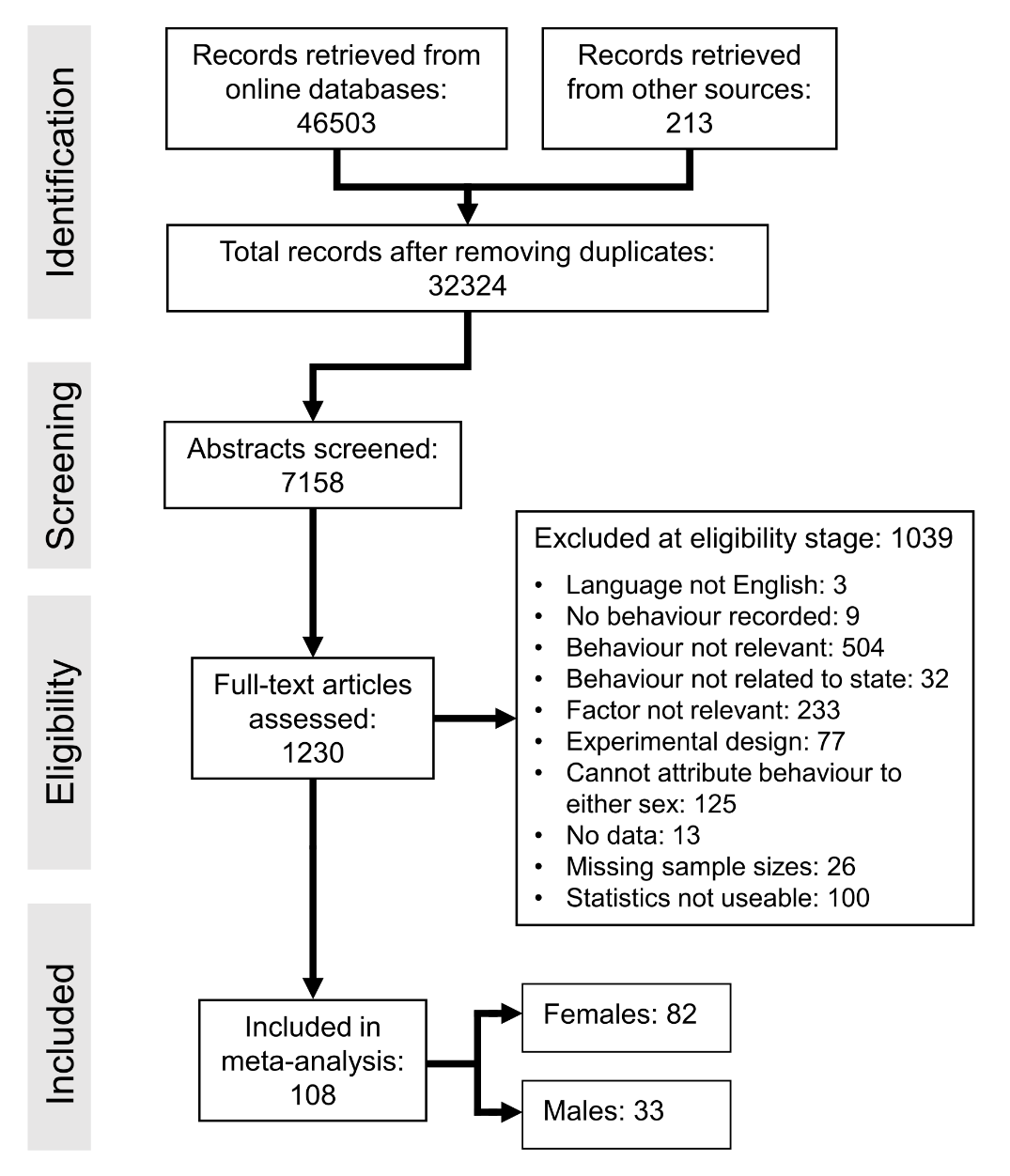
**

**Figure S1**. PRISMA diagram outlining the systematic literature search and study selection processes used to obtain the male and female data sets.


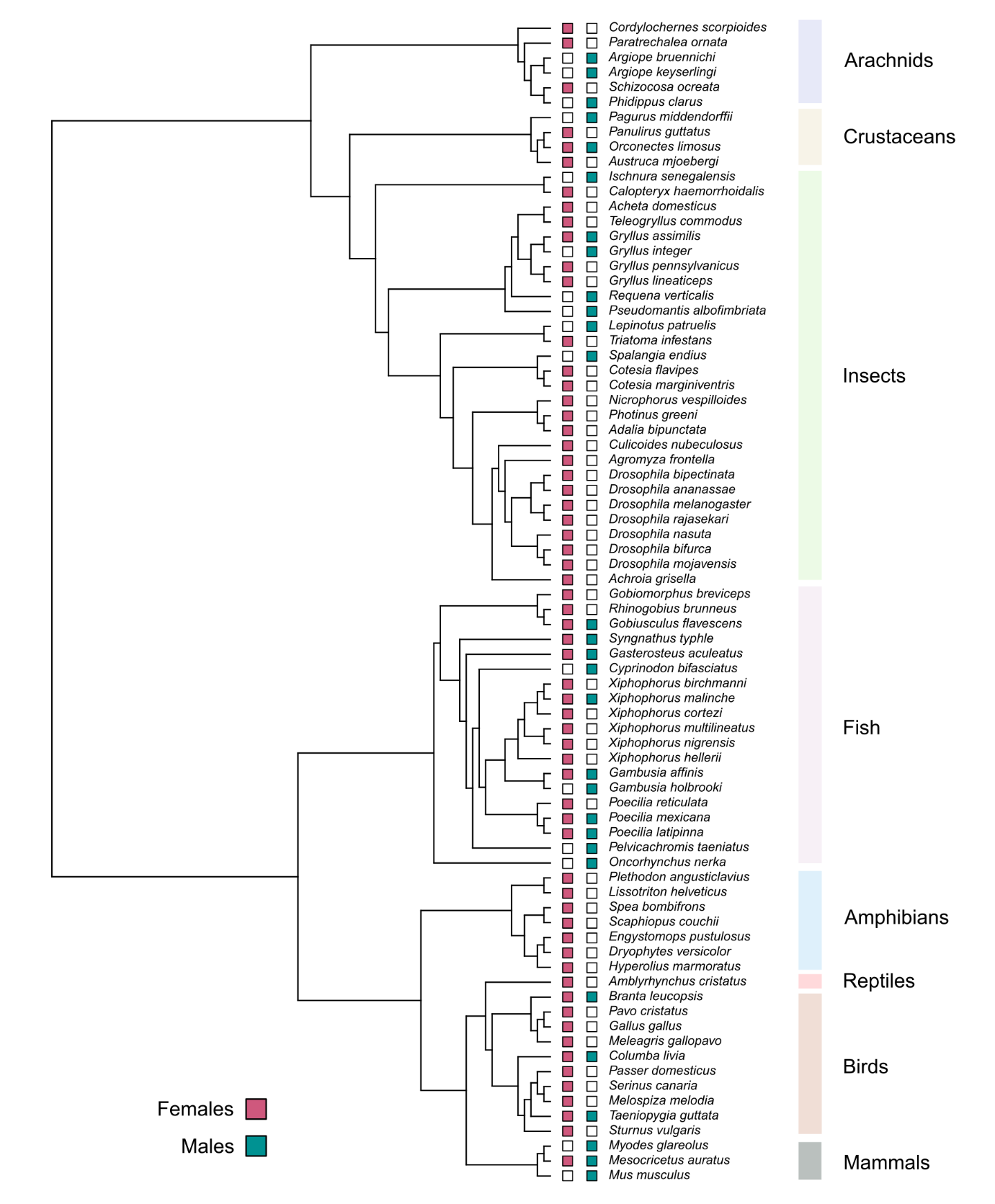


**Figure S2**. Phylogenetic relationships among the 78 species present in the study. Species with data for females are highlighted by a red box, and species with data for males by a green box. Note that the branch lengths are not time-calibrated. Instead, all branch lengths were set to one and then standardised using Grafen’s method.

**Sensitivity analysis: data type**

I calculated effect sizes (correlations) using one of four types of data:

1. Correlations (N= 34 for females and 19 for males)
2. Statistical tests comparing two groups (N= 26 for females and 5 for males)
3. Means and variances for two groups (N= 58 for females and 17 for males)
4. Frequency data (N= 22 for females and 18 for males)

As a form of sensitivity analysis, I tested whether the average correlation differed amongst these four data types, excluding directionless effect sizes. To do this I ran a meta-regression model with Zr as the response variable, data type as a single fixed factor, and phylogeny, species, study ID, and observation ID as random factors. I ran this model separately for males and females. Data type had no significant effect on the mean effect size for females (*Q_M_*= 1.57, *P*= 0.67, *k*= 140, marginal *R^2^*= 0.02) or males (*Q_M_*= 2.1, *P*= 0.55, *k*= 59, marginal *R^2^*= 0.04).

**Table S1**. Effect size estimates for different subsets of the female dataset. Mean effect size estimates (r), 95% confidence intervals, and 95% prediction intervals for each moderator category were obtained using a minus-intercept meta-regression, performed separately for each moderator.

| Factor | Level | Effect sizes | Studies | Species | Mean r | Lower 95% CI | Upper 95% CI | Lower 95% PI | Upper 95% PI |
| --- | --- | --- | --- | --- | --- | --- | --- | --- | --- |
| Taxonomic group | Amphibian | 9 | 7 | 7 | 0.087 | -0.186 | 0.348 | -0.564 | 0.671 |
|  | Arachnid | 8 | 3 | 3 | -0.085 | -0.416 | 0.265 | -0.689 | 0.588 |
|  | Bird | 28 | 16 | 10 | 0.078 | -0.091 | 0.243 | -0.547 | 0.648 |
|  | Crustacean | 3 | 3 | 3 | 0.014 | -0.384 | 0.408 | -0.651 | 0.667 |
|  | Fish | 58 | 26 | 15 | 0.078 | -0.051 | 0.205 | -0.541 | 0.642 |
|  | Insect | 69 | 25 | 22 | 0.143 | 0.018 | 0.264 | -0.491 | 0.679 |
|  | Mammal | 3 | 1 | 1 | 0.273 | -0.325 | 0.715 | -0.559 | 0.831 |
|  | Reptile | 1 | 1 | 1 | 0.000 | -0.686 | 0.686 | -0.791 | 0.791 |
| State factor | Age | 26 | 14 | 11 | -0.044 | -0.195 | 0.109 | -0.600 | 0.541 |
|  | Attractiveness | 4 | 4 | 4 | -0.192 | -0.486 | 0.141 | -0.720 | 0.478 |
|  | Body size | 50 | 27 | 26 | 0.135 | 0.019 | 0.249 | -0.466 | 0.651 |
|  | Condition | 67 | 29 | 21 | 0.059 | -0.052 | 0.169 | -0.523 | 0.604 |
|  | Mated status | 16 | 10 | 10 | 0.089 | -0.100 | 0.272 | -0.515 | 0.634 |
|  | Parasite load | 16 | 11 | 10 | 0.333 | 0.144 | 0.498 | -0.306 | 0.765 |
| State variation | Manipulated | 72 | 35 | 24 | 0.092 | -0.014 | 0.196 | -0.517 | 0.639 |
|  | Natural | 107 | 54 | 48 | 0.095 | 0.008 | 0.180 | -0.513 | 0.639 |
| Courter interaction | None | 80 | 39 | 31 | 0.074 | -0.031 | 0.177 | -0.533 | 0.630 |
|  | No physical | 28 | 17 | 11 | 0.095 | -0.065 | 0.250 | -0.525 | 0.649 |
|  | Full interaction | 71 | 26 | 26 | 0.121 | -0.002 | 0.240 | -0.500 | 0.660 |

**Table S2**. Results of pairwise contrasts between the six state factors for the female dataset (k= 175). Z values are presented below the diagonal, and P value above. Significant differences are highlighted in grey.

|  | Age | Body size | Condition | Mating status | Parasite load |
| --- | --- | --- | --- | --- | --- |
| Age |  | 1 | 1 | 1 | 0.19 |
| Body size | 1.38 |  | 1 | 1.00 | 0.56 |
| Condition | 0.92 | -0.97 |  | 1 | 0.15 |
| Mated status | 1.03 | -0.44 | 0.27 |  | 0.56 |
| Parasite load | 2.31 | 1.76 | 2.43 | 1.81 |  |

**Table S3**. Effect size estimates for different subsets of the male dataset. Mean effect size estimates (r), 95% confidence intervals, and 95% prediction intervals for each moderator category were obtained using a minus-intercept meta-regression, performed separately for each moderator.

| Factor | Level | Effect sizes | Studies | Species | Mean r | Lower 95% CI | Upper 95% CI | Lower 95% PI | Upper 95% PI |
| --- | --- | --- | --- | --- | --- | --- | --- | --- | --- |
| Taxonomic group | Arachnid | 7 | 3 | 3 | -0.079 | -0.831 | 0.775 | -0.931 | 0.907 |
|  | Bird | 6 | 3 | 3 | 0.079 | -0.765 | 0.823 | -0.904 | 0.929 |
|  | Crustacean | 2 | 2 | 2 | 0.200 | -0.728 | 0.869 | -0.885 | 0.947 |
|  | Fish | 29 | 14 | 11 | 0.369 | -0.553 | 0.885 | -0.812 | 0.957 |
|  | Insect | 18 | 8 | 7 | 0.145 | -0.676 | 0.806 | -0.873 | 0.927 |
|  | Mammal | 9 | 3 | 3 | 0.016 | -0.801 | 0.812 | -0.918 | 0.923 |
| State factor | Age | 1 | 1 | 1 | 0.217 | -0.403 | 0.701 | -0.623 | 0.825 |
|  | Attractiveness | 6 | 4 | 4 | 0.471 | 0.079 | 0.737 | -0.298 | 0.870 |
|  | Body size | 29 | 17 | 15 | 0.087 | -0.282 | 0.433 | -0.607 | 0.706 |
|  | Condition | 14 | 6 | 6 | 0.071 | -0.314 | 0.435 | -0.623 | 0.702 |
|  | Mated status | 17 | 9 | 8 | 0.154 | -0.224 | 0.492 | -0.564 | 0.740 |
|  | Parasite load | 4 | 2 | 2 | 0.335 | -0.168 | 0.699 | -0.477 | 0.838 |
| State variation | Manipulated | 25 | 11 | 11 | 0.133 | -0.124 | 0.373 | -0.465 | 0.647 |
|  | Natural | 46 | 24 | 22 | 0.161 | -0.077 | 0.383 | -0.436 | 0.660 |
| Courter interaction | None | 22 | 11 | 10 | 0.141 | -0.134 | 0.397 | -0.472 | 0.663 |
|  | No physical | 14 | 7 | 7 | 0.112 | -0.175 | 0.382 | -0.499 | 0.649 |
|  | Full interaction | 35 | 15 | 14 | 0.173 | -0.079 | 0.404 | -0.439 | 0.675 |


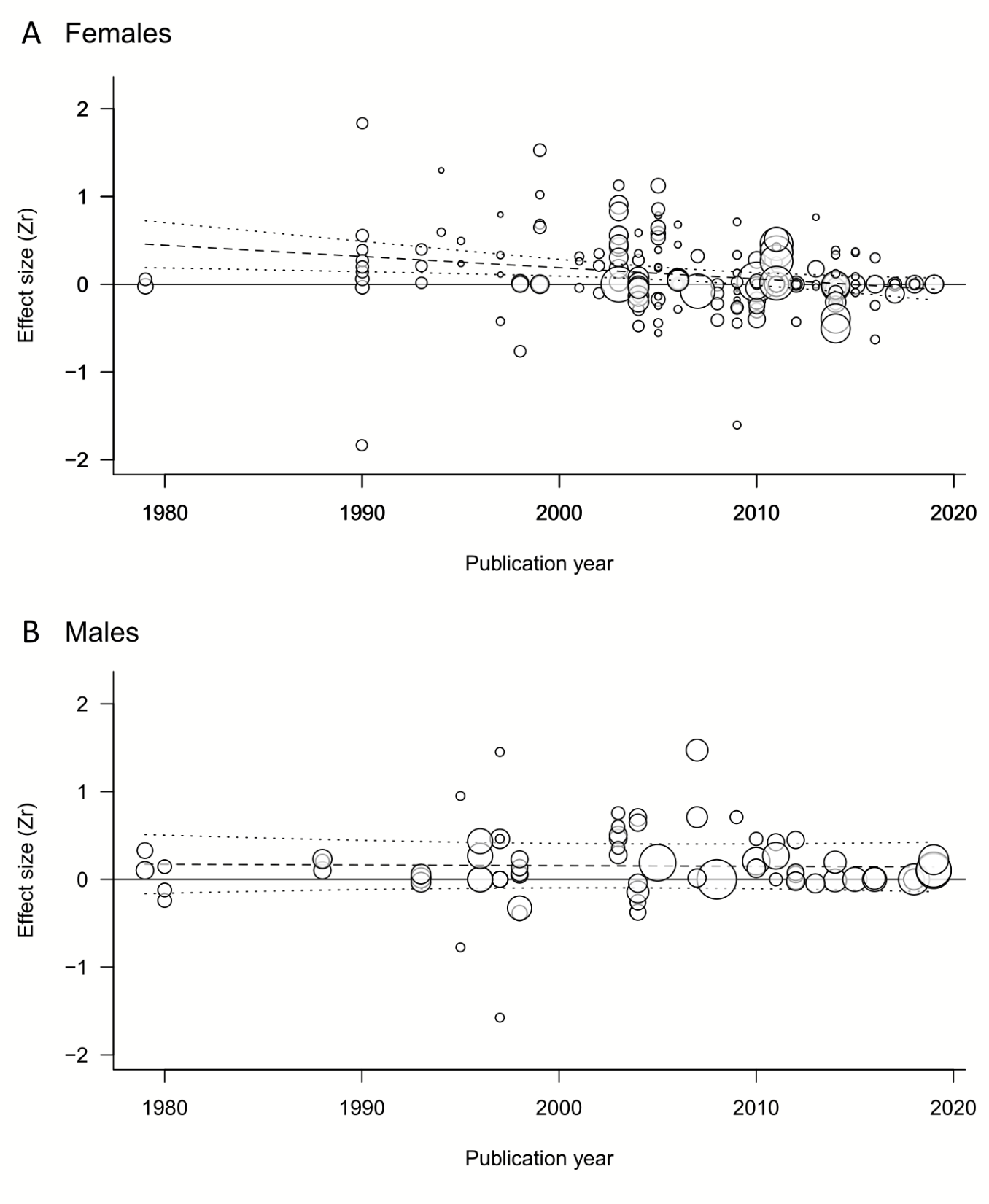


**Figure S3**. The relationship between study publication year and the effect size (Fisher’s z correlation between choosiness and individual state) for A. females (k= 179), and B. males (k= 71). The dashed and dotted lines show the mean estimate and 95% confidence intervals from the publication bias meta-regression model. Points are scaled by the inverse standard error of the estimate.


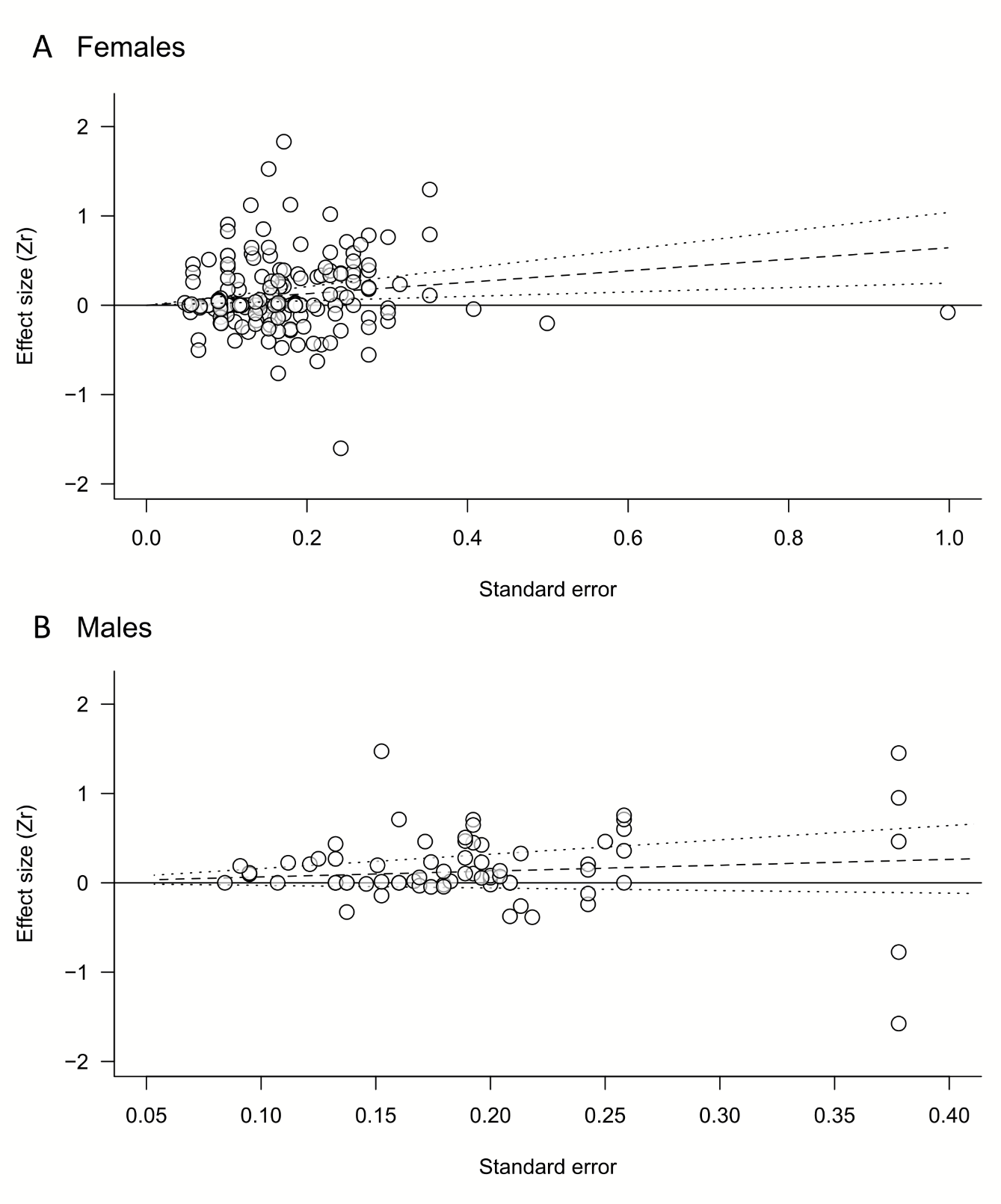


**Figure S4**. The relationship between the size of an effect (Fisher’s z correlation between choosiness and individual state) and its standard for A. females (k= 179), and B. males (k= 71). The dashed and dotted lines show the mean estimate and 95% confidence intervals from the publication bias meta-regression model.
